# The dissociation of semantically congruent and incongruent cross-modal effects on the visual attentional blink

**DOI:** 10.3389/fnins.2023.1295010

**Published:** 2023-12-14

**Authors:** Song Zhao, Yuxin Zhou, Fangfang Ma, Jimei Xie, Chengzhi Feng, Wenfeng Feng

**Affiliations:** ^1^Department of Psychology, School of Education, Soochow University, Suzhou, China; ^2^Research Center for Psychology and Behavioral Sciences, Soochow University, Suzhou, China

**Keywords:** attentional blink, cross-modal integration, audiovisual semantic congruency, congruent sound, ERPs

## Abstract

**Introduction:**

Recent studies have found that the sound-induced alleviation of visual attentional blink, a well-known phenomenon exemplifying the beneficial influence of multisensory integration on time-based attention, was larger when that sound was semantically congruent relative to incongruent with the second visual target (T2). Although such an audiovisual congruency effect has been attributed mainly to the semantic conflict carried by the incongruent sound restraining that sound from facilitating T2 processing, it is still unclear whether the integrated semantic information carried by the congruent sound benefits T2 processing.

**Methods:**

To dissociate the *congruence*-induced benefit and *incongruence*-induced reduction in the alleviation of visual attentional blink at the behavioral and neural levels, the present study combined behavioral measures and event-related potential (ERP) recordings in a visual attentional blink task wherein the T2-accompanying sound, when delivered, could be semantically neutral in addition to congruent or incongruent with respect to T2.

**Results:**

The behavioral data clearly showed that compared to the neutral sound, the congruent sound improved T2 discrimination during the blink to a higher degree while the incongruent sound improved it to a lesser degree. The T2-locked ERP data revealed that the early occipital cross-modal N195 component (192–228 ms after T2 onset) was uniquely larger in the congruent-sound condition than in the neutral-sound and incongruent-sound conditions, whereas the late parietal cross-modal N440 component (400–500 ms) was prominent only in the incongruent-sound condition.

**Discussion:**

These findings provide strong evidence that the modulating effect of audiovisual semantic congruency on the sound-induced alleviation of visual attentional blink contains not only a late incongruence-induced cost but also an early congruence-induced benefit, thereby demonstrating for the first time an unequivocal congruent-sound-induced benefit in alleviating the limitation of time-based visual attention.

## 1 Introduction

Due to limited attentional resources, we often lose some information that needs to be processed in complex and rapidly changing environments. However, recent studies have found that presenting information in multiple sensory modalities can facilitate the allocation of attention toward our targets (Van der Burg et al., 2011; Mastroberardino et al., 2015; Lunn et al., 2019; Turoman et al., 2021). One of the most typical illustrations of this facilitation is the sound-induced alleviation of visual attentional blink (Olivers and Van der Burg, 2008). The attentional blink refers to a phenomenon in which we are usually unable to recognize the second of two visual targets (T1 and T2) if they are presented within a short interval of approximately 200–500 ms (Raymond et al., 1992). However, when a task-irrelevant and uninformative auditory tone is delivered synchronously with T2, the attentional blink will be greatly alleviated, which is evidenced by improved T2 discrimination during the short T1-to-T2 interval (Olivers and Van der Burg, 2008; Kranczioch and Thorne, 2013, 2015; Kranczioch et al., 2018; Wang et al., 2022). Interestingly, although the attentional blink *per se* is well accepted as a suppression occurring late during the post-perceptual stage of processing (for reviews, see Martens and Wyble, 2010; Zivony and Lamy, 2022), the electrophysiological correlates of the auditory-induced alleviation of attentional blink have been shown to occur early during the perceptual stage (Kranczioch and Thorne, 2015).

However, the use of simple meaningless tones in the aforementioned studies on the auditory-induced alleviation of attentional blink does not allow exploration of the contribution of high-level audiovisual integration based on semantic relevance besides low-level integration based on audiovisual temporal correspondence. Using real-life sounds that could be semantically congruent or incongruent with T2 (e.g., when T2 was an image of a dog, a semantically congruent sound would be a dog bark and a semantically incongruent sound would be a car beep or drumbeat), a recent study explored the contribution of audiovisual semantic congruency (Zhao et al., 2021). It was found that although both semantically congruent and incongruent sounds improved T2 discrimination during the attentional blink interval, the semantically incongruent sound resulted in a smaller boost. More importantly, the event-related potential (ERP) data presented by Zhao et al. (2021) showed that both semantically congruent and incongruent audiovisual T2s elicited an occipitally distributed early cross-modal ERP component (N195, ~200 ms after T2 onset) with equal amplitude, whose occurrence has been thought to reflect the influence of auditory signals on early discriminative processing in the visual cortex (Giard and Peronnet, 1999; Molholm et al., 2004; Stekelenburg and Vroomen, 2005; Teder-Sälejärvi et al., 2005; Kaya and Kafaligonul, 2019). In contrast, only the semantically incongruent audiovisual T2 elicited a parietally distributed late cross-modal negativity (N440, ~400 ms after T2 onset), which is similar to the N400 component sensitive to semantic conflict (Kutas and Hillyard, 1980; Nieuwland et al., 2020). Accordingly, it was concluded that: (1) consistent with prior research (i.e., Kranczioch and Thorne, 2015), the auditory-induced alleviation of attentional blink has an early locus of facilitation; (2) the smaller T2 discrimination enhancement in the incongruent-sound relative to congruent-sound condition (i.e., the modulation of audiovisual semantic congruency) stems mainly from the semantic conflict carried by the incongruent sound restraining that sound from facilitating T2 processing at a late stage (Zhao et al., 2021).

The auditory-induced alleviation of visual attentional blink and the further modulation of semantic congruency are clear manifestations of the influences of spatiotemporal-based and semantic-based audiovisual integration on attentional state, respectively. Thus, investigating their psychophysiological mechanisms offers an excellent opportunity for researchers in the fields of multisensory integration and attention to unravel how low-level and high-level multisensory processes interplay to affect attention in general. Currently, there are two crucial questions regarding their psychophysiological mechanisms that need to be addressed. First, previous studies that manipulated audiovisual semantic congruency during the attentional blink (Adam and Noppeney, 2014; Zhao et al., 2021, 2022) did not include a condition wherein T2 is accompanied by a semantically neutral sound (e.g., a meaningless tone). Thus, it is still unclear whether the behavioral audiovisual semantic congruency effect consists of an additional *congruence*-induced benefit besides the aforementioned *incongruence*-induced cost. The existence of such a congruence-induced benefit would be reflected by a greater enhancement in the accuracy of T2 when accompanied by congruent sounds than when accompanied by neutral sounds, as inspired by a growing number of studies that have isolated beneficial effects originating purely from semantically congruent audiovisual integration (e.g., Hsiao et al., 2012; Heikkilä and Tiippana, 2016; Heikkilä et al., 2017; Nash et al., 2017; Xi et al., 2020; Du et al., 2022). Similarly, a pure incongruence-induced cost would be indexed by a smaller enhancement in the accuracy of T2 when accompanied by incongruent sounds than when accompanied by neutral sounds. Second, although Zhao et al. (2021) found that the early cross-modal ERP over the occipital scalp was independent of audiovisual semantic congruency, a subsequent study using a modified paradigm revealed that this neural activity was more pronounced in response to semantically congruent than incongruent audiovisual T2s (Zhao et al., 2022). The latter finding raises a possibility that the additional congruence-induced benefit may not only exist but also occur at an early stage of processing. However, due to the lack of a condition wherein T2 is paired with a meaningless sound, it remains to be determined whether such an early ERP difference signifies a congruence-induced benefit, an incongruence-induced cost, or both.

To dissociate at the behavioral and neural levels the congruence-induced benefit and incongruence-induced cost in the effect of audiovisual semantic congruency on the sound-induced alleviation of visual attentional blink, the current study combined behavioral measures and ERP recordings within the sound-accompanying visual attentional blink paradigm (Zhao et al., 2021), with the inclusion of the crucial condition wherein a meaningless tone was synchronous with T2 during the attentional blink. Thus, when accompanying T2, the sound could be semantically congruent, incongruent, or neutral with respect to T2. The task required participants to identify, as precisely as possible, T1 and T2 that were embedded in a rapid serial visual presentation (RSVP) stream at the end of each trial, while ignoring all accompanying sounds if delivered. Behaviorally, we found that the auditory-induced T2 discrimination enhancement during the blink was greater when congruent sounds accompanied T2 than when neutral sounds accompanied T2, and was greater when neutral sounds accompanied T2 than when incongruent sounds accompanied T2. Our ERP data showed that the occipitally distributed early cross-modal interaction N195 was larger in the congruent-sound condition than in the neutral-sound and incongruent-sound conditions, but the results in the latter two conditions did not differ. In contrast, the late cross-modal interaction N440 was elicited only in the incongruent-sound condition. These findings provide the first direct evidence to date that the modulating effect of audiovisual semantic congruency on the sound-induced alleviation of visual attentional blink consists of not only a late-occurring *incongruence*-induced cost but also a *congruence*-induced benefit at an early stage of visual discrimination.

## 2 Methods and materials

### 2.1 Participants

Thirty-seven healthy subjects participated in the current experiment. Data from three participants were excluded either because they withdrew from the experiment or due to excessive electroencephalogram (EEG) artifacts (> 40%), leaving the data of 34 subjects (20 females and 14 males; age range 18–26 years, mean age 20.2 years; all right-handed) for further analysis. The sample size was determined according to the sample size in the Zhao et al. (2021) study (*n* = 34) where a similar experimental paradigm was employed, as well as an *a priori* power analysis based on our pilot behavioral experiment. This pilot experiment found that the repeated-measures ANOVA with a single factor of audiovisual combination (VAcon, VAincon, VAneut) performed on the sound-induced T2 accuracy enhancement had a significant main effect, and the effect size *η^2^_p_* was 0.24. By entering this effect size into the software MorePower 6.0.4 (Campbell and Thompson, 2012), our power analysis showed that in the formal experiment, at least 18 participants should be recruited to obtain a power of 0.8 when finding such a significant main effect. All subjects verbally reported normal or corrected-to-normal visual acuity as well as normal hearing, and could easily identify the object categories (dogs, cars, and drums) of all visual and auditory stimuli used in the experiment. In accordance with the Declaration of Helsinki, written informed consent was obtained from all subjects before their participation, as approved by the Institutional Review Board of Soochow University.

### 2.2 Apparatus, stimuli, and design

The experiment was conducted in a dark and sound-attenuated room. Stimulus presentation was programmed using the software Presentation (version 18.0, NeuroBehavioral Systems, Inc.). Visual stimuli were presented on a 27-in. LCD monitor (ASUS PG279Q, resolution 1920 × 1,080, refresh rate 120 Hz) on which the background color was set to gray (RGB: 128, 128, 128). Auditory stimuli were delivered by a pair of loudspeakers (HiVi X3) positioned on the left and right sides of the monitor symmetrically. The horizontal and vertical distances between each of the loudspeakers and the center of the monitor were 21.5° and 0°, respectively. Subjects sat in front of the monitor with a viewing distance of approximately 80 cm, and were instructed to keep their eyes fixated on a red cross (RGB: 255, 0, 0; 0.3° × 0.3° in size), which was displayed at the center of the screen throughout each trial.

The visual stimuli consisted of 48 black-and-white line drawings (each 5.6° × 4.5°), including 30 unique drawings of houses used as distractors, nine unique drawings (three clothes, three cups, and three flowers) used as the first target (T1), and the remaining nine unique drawings (three dogs, three cars, and three drums) used as the second target (T2). The line drawings for T1 and T2 were from two non-overlapping sets in order to avoid priming (Koelewijn et al., 2008) or repetition blindness (Kanwisher, 1987). The auditory stimuli comprised nine unique object sounds (three dog barks, three car beeps, and three drumbeats) and one 1,000-Hz pure tone. They were all stereo, 200 ms in duration (with 20-ms rise and fall ramps), and approximately 75 dB in intensity at subjects’ ears when delivered by the loudspeakers. The line drawings and object sounds were the same as those used in the Zhao et al. (2021) study, and were also rated in terms of representativeness by an independent group of participants (see Supplementary materials for details). The pure tone was used as a semantically neutral sound because it was meaningless, carrying no semantic information, and previous studies on the meaningless-sound-induced alleviation of visual attentional blink (Olivers and Van der Burg, 2008; Kranczioch and Thorne, 2013, 2015; Kranczioch et al., 2018; Wang et al., 2022) also used pure tones to represent semantically neutral sounds (for further discussion about this issue, see Section 4).

It is also noteworthy that three different identities were arranged for each object category for two reasons. First, it could ensure that when participants were required to report the exact identities of T1 and T2, it was reasonably difficult for the task to induce a basic attentional blink at a short T1-to-T2 lag. Second, it could further prevent participants, to a certain degree, from guessing T2 based on what they heard. For example, even when T2 was a dog drawing and the synchronous sound was a dog bark, this sound was uninformative regarding which of the three dog drawings was the presented T2.

**Figure 1:**
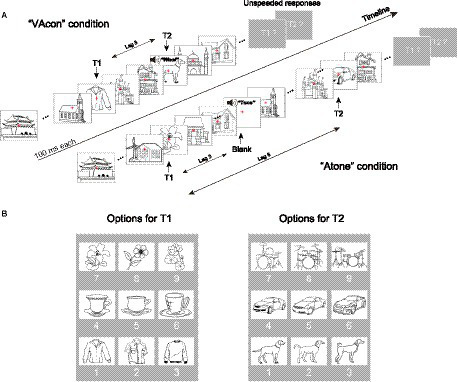
**(A)** Schematic illustrations of the 10-Hz RSVP streams in the “VAcon” condition (top left) and the “Atone” condition (bottom right), respectively. In a given RSVP stream, T2 appeared randomly and equiprobably at the third (lag 3) or eighth (lag 8) position after T1. When T2 was presented at lag 3, a semantically congruent object sound, a semantically incongruent object sound, a semantically neutral sound (i.e., a pure tone) or no sound was delivered synchronously with T2 (labeled as VAcon, VAincon, VAneut and V conditions, respectively). When T2 was presented at lag 8, the visual stimulus at lag 3 could be either a distractor (labeled as V_lag8 condition) or a blank drawing. In the latter case, an object sound, a pure tone or no sound was delivered synchronously with the blank drawing (labeled as Aobject, Atone and N conditions, respectively). These eight conditions were presented with equal probability. The task for participants was to discriminate sequentially the exact identities of T1 and T2 without time limit after each RSVP stream, while ignoring all sounds. **(B)** Nine options for T1 and another nine options for T2, along with their corresponding button numbers.

The experiment included 27 blocks; each block contained 64 trials, so each subject needed to complete a total of 1,728 trials. Specifically, each trial started with a red cross being presented alone for 1,000 ms, followed by a centrally presented RSVP stream (Figure 1A). Each RSVP stream comprised 17 distinct line drawings, containing two target drawings (T1 and T2) and 15 distractor drawings, with the latter ones being randomly selected from the above-mentioned 30 drawings of houses. Each drawing in the RSVP stream was presented for 100 ms, and was presented at the moment when the previous drawing disappeared. T1 could be a randomly selected drawing from nine drawings consisting of three clothes, three cups, and three flowers (see the left of Figure 1B), and was presented randomly from the third to fifth positions in the RSVP stream. T2 could be one of the remaining nine drawings (i.e., three dogs, three cars, and three drums; Figure 1B, right), and was presented in either the third or the eighth position after T1 (i.e., at lag 3 or lag 8) with equal probability. When T2 was presented at lag 3, it could be presented alone (labeled as “**V**” [visual-only] condition), synchronously with an object sound that was semantically congruent (labeled as “**VAcon**” [visual paired with congruent auditory] condition; e.g., a dog drawing with a dog bark; Figure 1A, top left), synchronously with an object sound that was semantically incongruent (labeled as “**VAincon**” [visual paired with incongruent auditory] condition; e.g., a dog drawing with a car beep), or synchronously with the aforementioned pure tone that was semantically neutral (labeled as “**VAneut**” [visual paired with neutral auditory] condition). Note that the wording “synchronously with” means “at the same onset time as.”

When T2 was presented at lag 8, either a distractor drawing or a white rectangle of the same size as the line drawings (i.e., a blank drawing) was presented at lag 3. The former case represented the standard visual-only lag 8 trials (labeled as “**V_lag8**” condition), which was set to check whether the attentional blink effect (i.e., much lower T2 discrimination accuracy at lag 3 than at lag 8) was reliably induced in the current study. In the latter case, the blank drawing at lag 3 could be presented synchronously with a randomly selected object sound from the nine object sounds (labeled as “**Aobject**” [auditory-object-only] condition), synchronously with the pure tone (labeled as “**Atone**” [auditory-tone-only] condition; Figure 1A, bottom right), or presented alone without any sounds (labeled as “**N**” [no-stimulus] condition). The three conditions were set in order to isolate ERP components associated with cross-modal interactions when semantically congruent, incongruent, and neutral sounds, respectively, were presented synchronously with T2s at lag 3 (see Section 2.4 for details). Note that in the Aobject condition, the object sound at lag 3 could be either congruent or incongruent with the subsequent T2 at lag 8 with equal probability, in order to counterbalance any forms of cross-modal semantic priming.

The above *eight* conditions (i.e., V, VAcon, VAincon, VAneut, Aobject, Atone, N, and V_lag8) were presented with equal probability (each 12.5%/216 trials) in a randomized order. The task for participants was to identify T1 and T2 as precisely as possible with no time limit by pressing buttons on a keyboard number pad with their right hands at the end of each RSVP stream while ignoring all sounds. The optional drawings for T1 and T2 and their corresponding button numbers were shown to the participants when they made their responses (see Figure 1B). Only when a T1 was precisely identified would this response be coded as a correct T1 identification, and the same rule applied to the definition of T2 identification. In terms of behavioral data analysis, on the basis of classic attentional blink studies (e.g., Raymond et al., 1992), the crucial T2 accuracy was calculated as the percentage of correct T2 identifications under the premise of correct T1 identifications. To prevent participants from guessing T2 identities based on the object sounds in the VAcon, VAincon, and Aobject conditions, they were explicitly informed that the object sounds’ semantic information was irrelevant to T2 identities. The button press for T2 then triggered the next trial. Participants were encouraged to have a rest between blocks in order to relieve fatigue.

It is worth mentioning that the experiment did not design corresponding VA conditions for T2s at lag 8 because the current study was aimed at investigating the effects of sounds on T2 discrimination *during* the attentional blink (i.e., at lag 3) rather than *outside* the blink (i.e., at lag 8). Furthermore, the current design allowed the acquisition of as many lag 3 trials as possible without unnecessarily prolonging the experiment, thus minimizing the fatigue effect (cf., Maier and Rahman, 2018) and ensuring acceptable signal-to-noise ratios for T2-locked ERP difference waveforms during the blink (see Section 2.4 for details). Importantly, a couple of recent studies have shown that both the effect of an object sound and the effect of a tone on T2 discrimination were negligible outside the blink (Zhao et al., 2021; Wang et al., 2022), which further indicates that presenting sounds synchronously with T2s at lag 8 would be redundant in the current study.

### 2.3 Electrophysiological recording and preprocessing

The EEG of participants who performed the task was recorded continuously through a SynAmps2 amplifier (NeuroScan, Inc.) and a custom-built 64-electrode elastic cap. The electrodes on the cap were located on the basis of a modified 10–10 system montage (for details, see Zhao et al., 2022). Two additional electrodes, AFz and M1 (left mastoid), served as the ground and reference electrodes during data acquisition, respectively. The horizontal electrooculogram (HEOG) elicited by leftward and rightward eye movements was recorded through a pair of bipolar electrodes located on the left and right outer canthi. The vertical electrooculogram (VEOG) elicited by vertical eye movements and blinks was recorded by another pair of bipolar electrodes positioned above and below the left eye. The impedances of all electrodes were maintained under 5 kΩ. The online EEG and EOG signals were filtered by a band-pass filter of 0.05–100 Hz and digitized at a sampling rate of 1,000 Hz. Recordings were carried out using the software SCAN (version 4.5, NeuroScan, Inc.).

In offline preprocessing, the continuous EEG signals were first down-sampled to 500 Hz, and then low-pass filtered (half-amplitude cutoff = 33.75 Hz, transition band width = 7.5 Hz) using a zero-phase shifted (two-pass forward and reverse), Hamming-windowed sinc FIR filter to attenuate high-frequency noise triggered by muscle activities or external electrical sources. The filtered EEG data were re-referenced to the average of the left and right mastoid (M1 and M2) electrodes. The re-referenced EEG signals in all but the V_lag8 condition were split into 600-ms epochs time-locked to the lag 3 position (for V, VAcon, VAneut, and VAincon conditions, time-locked to T2 onset; for Atone, Aobject, and N conditions, time-locked to the blank drawing onset; see Figure 1A) with a 100-ms pre-lag 3 baseline. In terms of artifact correction, independent component analysis (ICA) was applied to these EEG epochs to identify and remove independent components (ICs) corresponding to common EEG artifacts such as horizontal eye movements and eye blinks (Delorme and Makeig, 2004). On average, 3.91 (*SE* = 0.42) such ICs were removed. The post-ICA epochs were first baseline corrected, and epochs contaminated by residual artifacts were then discarded on the basis of a threshold of ±75 μV. Participants were excluded from further analysis if more than 40% of the epochs were lost after artifact rejection (three participants were excluded; see Section 2.1). In accordance with prior EEG studies using attentional blink paradigms (e.g., Vogel et al., 1998; Zhao et al., 2021, 2022), only trials (epochs) with correct T1 identification were further analyzed, hence leaving an average of 175.96 (*SE* = 1.84) valid epochs per condition. The remaining valid epochs were averaged separately for each condition to obtain the corresponding ERP waveforms. The EEG preprocessing and subsequent ERP analysis were conducted using the EEGLAB toolbox (Delorme and Makeig, 2004) in conjunction with custom-built MATLAB scripts (The MathWorks, Inc.).

### 2.4 ERP data analysis

To reveal brain activity responsible for the effects of different sounds on T2 identification during the attentional blink, the present study calculated cross-modal difference waveforms separately for the VAcon, VAincon, and VAneut conditions. In principle, the cross-modal difference waveforms are calculated by subtracting the sum of ERPs to unimodal visual and auditory stimuli from ERPs to bimodal audiovisual stimuli; statistically significant positive or negative deflections (as compared with 0) in the difference waveforms have been considered as neural activities associated with audiovisual cross-modal interactions (Giard and Peronnet, 1999; Molholm et al., 2004; Bonath et al., 2007; Mishra et al., 2007; Brandwein et al., 2013; Kranczioch and Thorne, 2015; Zhao et al., 2018, 2020, 2021, 2022). Specifically, the current three cross-modal difference waveforms (labeled as “**VAcon_diff**,” “**VAincon_diff**” and “**VAneut_diff**”) were calculated according to the following:

(1) when T2 was paired with a semantically congruent sound: VAcon_diff = VAcon − (V + Aobject);
(2) when T2 was paired with a semantically incongruent sound: VAincon_diff = VAincon − (V + Aobject);
(3) when T2 was paired with a semantically neutral tone: VAneut_diff = VAneut − (V + Atone).

Notably, these calculations could balance out any ERP differences resulting from the inherent physical differences between the unimodal auditory elements in the VAcon/VAincon condition (i.e., object sounds) and those in the VAneut condition (i.e., pure tones), thereby allowing direct ERP comparisons among the three difference waveforms.

Prior to the above calculations, note that the lag 3-locked ERPs elicited in the N condition (in which a blank drawing appeared at lag 3 with no sound) were first subtracted from ERPs in each of the remaining six conditions (i.e., VAcon, VAincon, VAneut, V, Atone, and Aobject). These subtractions not only removed the distractor-elicited ERPs and left ERPs elicited purely by stimuli at lag 3 for each condition (cf., Vogel et al., 1998; Sergent et al., 2005; Luo et al., 2010, 2013; Kranczioch and Thorne, 2015; Maier and Rahman, 2018; Zhao et al., 2021, 2022), but also counterbalanced any pre-lag 3 anticipatory activities (e.g., CNV) common to all conditions that may lead to false detection of early cross-modal interactions when calculating the cross-modal difference waveforms (cf., Talsma and Woldorff, 2005; Bonath et al., 2007; Mishra et al., 2007; Van der Burg et al., 2011; Zhao et al., 2018, 2020, 2021). In addition, because in the Aobject, Atone, and N conditions the time epoch of interest (i.e., −100 to +500 ms relative to the blank drawing onset at lag 3) *preceded* the onset of T2 at lag 8, the isolated cross-modal ERPs during this epoch would not be confounded by T2-related ERPs at lag 8 (Zhao et al., 2022).

Subsequently, the spatiotemporal parameters for quantifying the main ERP components in the cross-modal difference waveforms were determined *a priori* according to those used in recent ERP studies of the cross-modal boost during attentional blink (Zhao et al., 2021, 2022). That is, the time windows and electrodes listed below were chosen because the current ERP components of interest have been found to be maximal over these time windows and electrodes in previous studies. This approach minimizes the problem of implicit multiple comparisons that could result in inflation of the Type I error rate, as recommended in recent ERP literature (Luck and Gaspelin, 2017). First, the occipital N195 component was measured as the average amplitude during 192–228 ms after T2 onset over three adjacent occipital electrodes O1, Oz, and O2. Second, the parietal N440 component was measured as the average amplitude during a time window of 400–500 ms after the onset of T2 over three neighboring parietal electrodes P1, Pz, and P2. The measurement window for the N440 here was broader than that used previously (i.e., 424–448 ms) because: (a) this component is seemingly reliable throughout 400–500 ms, as indicated by recent studies (Zhao et al., 2021, 2022); (b) using a broader window to measure slow ERPs like the N440 would better avoid the impact of EEG noise (Luck, 2014).

For statistical analysis, to explore the stage of processing at which the congruent-sound-induced contribution to the alleviation of attentional blink occurred, separate one-way repeated-measures ANOVAs with a factor of audiovisual combination (VAcon, VAincon, VAneut) were conducted on the amplitudes of each ERP component in the cross-modal difference waveforms. Only when the main effect of audiovisual combination was significant would pairwise comparisons be performed further by paired-samples *t*-tests, with the contrast between the VAcon and VAneut conditions being the primary focus. Furthermore, to check the presence/absence of each ERP component in each condition, separate one-sample *t*-tests were conducted between zero and the amplitude of each ERP wave in each of the three difference waveforms.

In the case of nonsignificant results, Bayesian statistics were further conducted to evaluate to what extent the null hypothesis could be true, using the software JASP^1^ with a default Cauchy scale value of 0.707. A Bayes factor (*BF_10_*) falling within 0.333–1.000 is considered as anecdotal evidence, within 0.100–0.333 as moderate evidence, and below 0.100 as strong evidence for the null hypothesis (Dienes, 2014; Wagenmakers et al., 2018).

## 3 Results

### 3.1 Behavioral data

**Figure 2:**
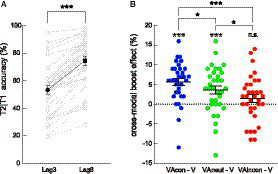
Behavioral results. **(A)** Mean accuracy of T2 discrimination (given T1 correct) plotted as a function of lag (lag 3, lag 8) for visual-only (i.e., V and V_lag8) trials. T2 discrimination accuracy on the lag 3 trials was significantly lower than that on the lag 8 trials. The single-subject data are depicted by scatter dots, the group-averaged data are marked by black symbols, and error bars represent ±1 SE (the same below). **(B)** Cross-modal boost effect at lag 3 (quantified by the VA-minus-V difference in T2|T1 accuracy) plotted as a function of audiovisual semantic congruency (congruent, neutral, incongruent). Compared to the VAneut condition, the cross-modal boost was larger in the VAcon condition and smaller in the VAincon condition. The symbols in white denote the significance of these cross-modal boost effects against zero. *: *p* < 0.05; **: *p* < 0.01; ***: *p* < 0.001; n.s., non-significant.

A paired *t*-test conducted on T2 discrimination accuracy (given correct T1 discrimination) in the visual-only lag 3 (i.e., V) and visual-only lag 8 (i.e., V_lag8) conditions showed that T2 performance was significantly worse at lag 3 than at lag 8 [V: 53.07 ± 3.20% (*M* ± *SE*, the same below); V_lag8: 74.38 ± 3.13%; *t*(33) = −11.794, *p* < 0.001, *Cohen’s d* = −2.023, 95% CI for *d* = [−2.608, −1.427]; Figure 2A], indicating that the experiment successfully triggered an attentional blink when T2 was presented at lag 3 (Raymond et al., 1992).

Afterwards, within lag 3 trials, T2 accuracy in the V condition was subtracted from T2 accuracy in each VA condition (i.e., VAcon, VAincon, and VAneut) to isolate the behavioral “cross-modal boost effect” for each of these conditions (Figure 2B). These difference measures were then subject to a repeated-measures ANOVA with a single factor of audiovisual combination (VAcon, VAincon, VAneut). The results showed that the main effect of audiovisual combination was significant [*F*(2, 66) = 9.776, *p* < 0.001, *η^2^_p_* = 0.229, 95% CI for *η^2^_p_* = [0.062, 0.374]]. Pairwise comparisons by paired *t*-tests first showed that the cross-modal boost effect was significantly larger in the VAcon condition (5.65 ± 0.88%) than in the VAincon condition [1.43 ± 0.97%; *t*(33) = 4.124, *p* < 0.001, *d* = 0.707, 95% CI for *d* = [0.326, 1.080]], replicating the effect of audiovisual semantic congruency on the cross-modal boost during attentional blink (Adam and Noppeney, 2014; Zhao et al., 2021, 2022). More importantly, the cross-modal boost effect was significantly larger in the VAcon condition [*t*(33) = 2.135, *p* = 0.040, *d* = 0.366, 95% CI for *d* = [0.016, 0.711]] and was significantly smaller in the VAincon condition [*t*(33) = −2.463, *p* = 0.019, *d* = −0.422, 95% CI for *d* = [−0.711, −0.068]] relative to that in the VAneut condition (3.66 ± 0.99%). Thus, with the addition of a condition in which T2 was accompanied by a semantically neutral sound, the current study clearly demonstrates that the modulation of audiovisual semantic congruency consists of not only an *incongruence*-induced cost that decreases the cross-modal boost of T2 discrimination as reported recently (Zhao et al., 2021) but also an additional *congruence*-induced benefit that increases the cross-modal boost.

In addition, to check the robustness of the above cross-modal boost effects *per se*, one-sample *t*-tests were conducted between each of these effects versus zero. The results yielded that the cross-modal boost during attentional blink was significant in both VAcon [*t*(33) = 6.388, *p* < 0.001, *d* = 1.095, 95% CI for *d* = [0.663, 1.517]] and VAneut [*t*(33) = 3.685, *p* = 0.001, *d* = 0.632, 95% CI for *d* = [0.259, 0.997]; Figure 2B] conditions, consistent with the findings of previous studies using real-life sounds (e.g., Zhao et al., 2021) and those using pure tones (e.g., Olivers and Van der Burg, 2008). In contrast, the boost did not reach significance in the VAincon condition [*t*(33) = 1.460, *p* = 0.154, *d* = 0.250, 95% CI for *d* = [−0.093, 0.590], *BF_10_* = 0.483], which is also in agreement with a more recent finding (Zhao et al., 2022), although the Bayes factor only provided anecdotal evidence for the null hypothesis. Note that the little-to-no cross-modal boost in the VAincon condition does not mean that no incongruence-induced cost existed because the incongruence-induced cost refers to the cross-modal boost being smaller in the VAincon condition than in the VAneut condition, as reported above.

### 3.2 ERP data

**Figure 3:**
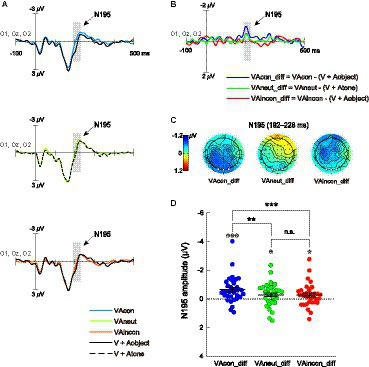
**(A)** Bisensory ERPs elicited by T2s paired with congruent sounds (VAcon), T2s paired with neutral sounds (VAneut) and T2s paired with incongruent sounds (VAincon), as well as the summed unisensory ERPs elicited by T2s and object sounds (V + Aobject) and T2s and pure tones (V + Atone), which were averaged over the electrodes O1, Oz and O2. The shaded areas on ERP waveforms depict the time window (192–228 ms) within which the mean amplitude of the N195 difference component was quantified for further comparisons. **(B)** N195 component revealed in the cross-modal difference waveform was uniquely larger for the VAcon condition than for the VAneut and VAincon conditions. **(C)** Scalp topographies of the N195 components revealed in each cross-modal difference waveform. The white dots on each scalp topography depict the electrodes O1, Oz, and O2 over which the N195 was measured. **(D)** Summary of the N195 mean amplitudes as a function of audiovisual semantic congruency (congruent, neutral, incongruent). The single-subject data are depicted by scatter dots, the group-averaged data are marked by black symbols, and error bars correspond to ±1 SE. The symbol “*” in white denotes a significant N195 amplitude against zero. *: *p* < 0.05; **: *p* < 0.01; ***: *p* < 0.001; n.s., non-significant.

**Figure 4:**
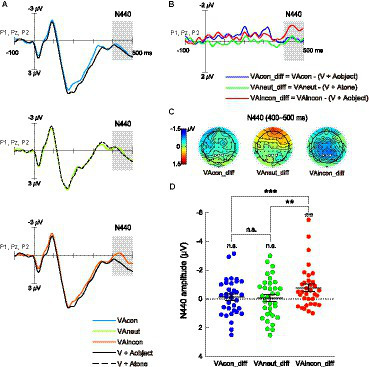
Same as Figure 3 but for the N440 difference component, which was significantly evoked solely in the VAincon condition. The ERP waveforms were averaged over the electrodes P1, Pz, and P2. The shaded areas on waveforms and the white dots on scalp topographies show the 400–500 ms time window and the three parietal electrodes where the N440 mean amplitude was quantified.

To extract brain activity responsible for the effects of different sounds on T2 discrimination during the attentional blink, cross-modal difference waveforms were calculated separately for the VAcon, VAincon, and VAneut conditions (see Section 2.4). For completeness, the bimodal ERPs elicited by T2-sound pairs (i.e., VAcon, VAincon, VAneut) and the summed unimodal ERPs elicited by T2s and sounds (i.e., [V + Aobject], [V + Atone]), which were ingredients of these cross-modal difference waveforms, are shown in Figure 3A (averaged waveforms over occipital electrodes O1, Oz, and O2) and Figure 4A (averaged waveforms over parietal electrodes P1, Pz, and P2).

#### 3.2.1 Early cross-modal N195 component

The first ERP component of interest in the difference waveforms was the early cross-modal negativity N195 over the occipital scalp (Figures 3B,C), which has been shown to underlie not only the cross-modal boost during attentional blink (Zhao et al., 2021) but also the further audiovisual semantic congruency effect (Zhao et al., 2022). A repeated-measures ANOVA with a single factor of audiovisual combination (VAcon_diff, VAincon_diff, VAneut_diff) was conducted on the N195 amplitudes, and the main effect was significant [*F*(2, 66) = 5.842, *p* = 0.005, *η^2^_p_* = 0.150, 95% CI for *η^2^_p_* = [0.030, 0.266]]. Pairwise comparisons showed that the N195 amplitude in the VAcon_diff waveform (−0.66 ± 0.16 μV) was significantly greater not only than that in the VAincon_diff waveform as expected [−0.28 ± 0.14 μV; *t*(33) = −3.420, *p* = 0.002, *d* = −0.587, 95% CI for *d* = [−0.947, −0.218]] but also than that in the VAneut_diff waveform [−0.30 ± 0.14 μV; *t*(33) = −3.052, *p* = 0.004, *d* = −0.523, 95% CI for *d* = [−0.879, −0.161]; Figure 3D]. However, the N195 amplitude did not differ between the VAincon_diff and VAneut_diff waveforms, with the Bayes factor providing moderate evidence for the null hypothesis [*t*(33) = 0.124, *p* = 0.902, *d* = 0.021, 95% CI for *d* = [−0.315, 0.357], *BF_10_* = 0.185]. In addition, one-sample *t*-tests against zero further showed that the N195 component was significantly elicited in each of the three difference waveforms [VAcon_diff: *t*(33) = −4.208, *p* < 0.001, *d* = −0.722, 95% CI for *d* = [−1.095, −0.339]; VAneut_diff: *t*(33) = −2.172, *p* = 0.037, *d* = −0.372, 95% CI for *d* = [−0.718, −0.022]; VAincon_diff: *t*(33) = −2.078, *p* = 0.046, *d* = −0.356, 95% CI for *d* = [−0.701, −0.007]; Figure 3D]. Taken together, the current N195 results indicate not only that the congruent-sound-induced benefit to T2 performance at lag 3 occurs at an early stage of visual discrimination, but also that the incongruent-sound-induced cost has not yet unfolded at that stage.

#### 3.2.2 Late cross-modal N440 component

The second ERP of interest in the difference waveforms was the late cross-modal negativity N440 over the parietal resign (Figures 4B,C), which has been shown to underlie the effect of audiovisual semantic congruency on the cross-modal boost during attentional blink (Zhao et al., 2021, 2022). The same one-way repeated-measures ANOVA conducted on the N440 amplitudes yielded a significant main effect of audiovisual combination [*F*(2,66) = 7.778, *p* = 0.001, *η^2^_p_* = 0.191, 95% CI for *η^2^_p_* = [0.038, 0.335]]. Pairwise comparisons revealed that the N440 amplitude in the VAincon_diff waveform (−0.78 ± 0.25 μV) was significantly greater not only than that in the VAcon_diff waveform as expected [−0.12 ± 0.22 μV; *t*(33) = −4.117, *p* < 0.001, *d* = −0.706, 95% CI for *d* = [−1.078, −0.325]] but also than that in the VAneut_diff waveform [−0.08 ± 0.24 μV; *t*(33) = −3.094, *p* = 0.004, *d* = −0.531, 95% CI for *d* = [−0.887, −0.168]; Figure 4D]. However, there was no difference in N440 amplitude between the VAcon_diff and VAneut_diff waveforms, with the Bayes factor providing moderate evidence for the nonsignificant result [*t*(33) = −0.216, *p* = 0.831, *d* = −0.037, 95% CI for *d* = [−0.373, 0.300], *BF_10_* = 0.188]. In fact, one-sample *t*-tests showed that the N440 amplitude was significant against zero only in the VAincon_diff waveform [*t*(33) = −3.130, *p* = 0.004, *d* = −0.537, 95% CI for *d* = [−0.893, −0.173]], but not in either the VAcon_diff waveform [*t*(33) = −0.568, *p* = 0.574, *d* = −0.097, 95% CI for *d* = [−0.434, 0.240], *BF_10_* = 0.213] or the VAneut_diff waveform [*t*(33) = −0.327, *p* = 0.746, *d* = −0.056, 95% CI for *d* = [−0.392, 0.281], *BF_10_* = 0.193; Figure 4D], with Bayes factors providing moderate evidence for the two nonsignificant results. These findings suggest that the incongruent-sound-induced cost, which was manifested as a smaller cross-modal boost in the VAincon relative to VAneut condition (Figure 2B), unfolds at a late stage of processing.

Further inspection of the single-subject data in Figure 3D and Figure 4D suggests that there might be outliers in the difference waveform datasets. To examine whether this is the case, for each of the six relevant ERP datasets (i.e., N195 and N440 amplitudes in the VAcon_diff, VAincon_diff, and VAneut_diff waveforms), we checked if there were single-subject amplitude values that fell outside three standard deviations from the grand-averaged mean amplitude. It was found that the largest N195 amplitude in the VAcon_diff dataset and the largest N440 amplitude in the VAincon_diff dataset fell outside this criterion, and the two outliers came from the same participant. Thus, we re-ran the aforementioned statistical tests for the ERP data after excluding this participant’s data. However, none of the ERP effects has changed. Therefore, for the purpose of data completeness, we retained the ERP results calculated based on the data of all 34 participants.

## 4 Discussion

Previous studies have found that the sound-induced T2 accuracy enhancement during visual attentional blink was smaller for semantically incongruent sounds than for semantically congruent sounds, and this weaker enhancement was attributed mainly to the semantic conflict carried by incongruent sounds reducing the ability of these sounds to facilitate T2 processing at a late stage (Zhao et al., 2021, 2022). However, it remains to be determined whether the integrated semantic information carried by congruent sounds benefits T2 processing, thereby contributing also to the congruent versus incongruent difference. The present ERP study dissociated the *congruence*-induced benefit (which would increase the cross-modal boost of T2 discrimination) and the *incongruence*-induced cost (which would decrease the cross-modal boost) by adding a crucial baseline condition in which a semantically neutral sound was presented synchronously with T2. The behavioral data clearly showed that compared to the neutral sounds, the incongruent sounds boosted T2 discrimination to a lesser degree, while the congruent sounds boosted T2 discrimination to a higher degree. These findings replicate the alleviation of visual attentional blink induced by meaningless sounds (Olivers and Van der Burg, 2008; Kranczioch and Thorne, 2013, 2015; Kranczioch et al., 2018; Wang et al., 2022) as well as the modulating effect of audiovisual semantic congruency (congruent vs. incongruent) on it (Adam and Noppeney, 2014; Zhao et al., 2021, 2022). More importantly, these findings provide the first evidence that the modulation of audiovisual semantic congruency is bidirectional, containing not only a cost induced by the incongruent sounds but also an additional benefit induced by the congruent sounds. This congruence-induced benefit is consistent with the additional gains of cross-modal integration induced by semantically congruent relative to semantically irrelevant audiovisual stimuli found in an accumulating number of studies (Hsiao et al., 2012; Heikkilä and Tiippana, 2016; Heikkilä et al., 2017; Nash et al., 2017; Xi et al., 2020; Du et al., 2022; Duarte et al., 2022; Maezawa et al., 2022), thereby highlighting the importance of including semantically unrelated audiovisual stimuli when dissociating the effects of semantically congruent and incongruent audiovisual integration.

It may be argued that the higher T2 discrimination accuracy induced by congruent object sounds than by neutral sounds might have resulted simply from a response bias. Specifically, despite the fact that the semantic content of the object sounds was completely uninformative of T2 identities (see Section 2.2), when hearing an object sound, participants might still be biased to choose one of the three line drawings corresponding to that sound (e.g., picking one of the three dog drawings just because a dog bark was delivered; see Figure 1B). In contrast, such response bias would not exist when they heard a neutral sound. Consequently, it is possible that participants might have chosen *the correct T2 object categories* (e.g., choosing one of the three dogs when T2 was a dog, regardless of whether the chosen one was identical to the presented one) more frequently in the congruent-sound condition relative to the neutral-sound condition. This inclination could further lead to *the correct T2 identities* (based on which our T2 accuracy was calculated) being hit on more trials in the former than in the latter condition, hence the observed higher T2 accuracy in the congruent-sound condition. However, it is strongly noteworthy that if this crucial effect was indeed merely driven by such response bias, we should further predict that there was no substantial difference in *the likelihood of choosing correct T2 identities relative to choosing correct T2 object categories* between the congruent- and neutral-sound conditions. Alternatively, if this effect was mainly driven, as we proposed, by the congruence-induced benefit to detailed discrimination of T2, that likelihood should be significantly higher in the congruent-sound condition than in the neutral-sound condition. Our *post hoc* analysis of that likelihood provides clear evidence for the latter prediction [VAcon: 65.96 ± 2.97% (*M* ± *SE*); VAneut: 62.83 ± 2.76%; *t*(33) = 2.058, *p* = 0.048, *d* = 0.353, 95% CI for *d* = [0.004, 0.697]]. Thus, although such response bias should be controlled more strictly in the future, the higher T2 discrimination accuracy in the congruent- than neutral-sound condition here is more likely to reflect a true congruence-induced benefit to T2 discrimination during the attentional blink. Indeed, this proposal is further supported by the concurrently recorded ERP data (see below).

Electrophysiologically, we first examined the early cross-modal difference component N195 over the occipital region (measured during 192–228 ms after T2 onset over the electrodes O1, Oz, and O2). This component has been shown to be the neural basis of the cross-modal boost of T2 discrimination accuracy during the attentional blink (Zhao et al., 2021). A recent follow-up study further found that this component was larger when T2 was paired with a semantically congruent sound than when T2 was paired with an incongruent sound (Zhao et al., 2022). However, the psychophysiological mechanisms reflected by such a congruent-minus-incongruent ERP difference were uncertain, because it might represent either a congruence-induced benefit, an incongruence-induced cost, or both. The current study provides direct evidence for the first possibility by showing that the N195 elicited in the congruent-sound condition was greater than the N195 elicited in the neutral-sound condition wherein only low-level audiovisual integration based on spatiotemporal correspondence was expected. In contrast, the N195 elicited in the incongruent-sound condition was not smaller than the N195 elicited in the neutral-sound condition. Importantly, given that cross-modal neural activities closely resembling the N195 component have been thought to reflect the influence of auditory signals on early discriminative processing in the visual cortex (Giard and Peronnet, 1999; Molholm et al., 2004; Stekelenburg and Vroomen, 2005; Teder-Sälejärvi et al., 2005; Kaya and Kafaligonul, 2019), the current N195 results demonstrate that the congruent-sound-induced semantic benefit to T2 processing during the attentional blink has already begun at an early stage of visual discrimination, whereas the incongruent-sound-induced cost has not yet unfolded at that stage.

It is worth mentioning that Zhao et al. (2021) did not observe a basic congruent-minus-incongruent difference in the N195 amplitude, whereas their follow-up study (Zhao et al., 2022) and the current study did. On closer inspection, we speculate that this discrepancy is most likely to be due to a subtle difference in experimental paradigm between the former and latter studies. In Zhao et al. (2021) paradigm, T2 was likely to be absent (i.e., substituted with a blank drawing) in a total of 40% of the RSVP streams (see their Figure 1C). However, in the paradigms of Zhao et al. (2022) and the current study, T2 was presented *de facto* in 100% of the RSVP streams, including those RSVP streams where a blank drawing had been presented (e.g., the current Atone condition illustrated in Figure 1A). Accordingly, the difference in T2 probability might have led observers to employ somewhat different top-down strategies in the processing of T2, thereby giving rise to the above-mentioned discrepancy in the presence/absence of the congruent-minus-incongruent N195 difference. Although more compelling evidence to support this speculation is required in the future, it is consistent with a series of prior findings which suggest that the audiovisual semantic congruency effect on the occipital ERP around 200 ms post-stimulus seems sensitive to top-down attentional allocation (Molholm et al., 2004; Yuval-Greenberg and Deouell, 2007; Sinke et al., 2014).

What is also noteworthy is that the N195 component was negative in polarity in Zhao et al. (2021) and the current study but was positive (hence labeled as P195) in Zhao et al. (2022). As discussed by Zhao et al. (2022), the polarity of similar occipitally distributed early cross-modal ERP difference components also varies from study to study (negative: e.g., Molholm et al., 2004, 2020; Brandwein et al., 2011, 2013; Kaya and Kafaligonul, 2019; positive: e.g., Giard and Peronnet, 1999; Molholm et al., 2002; Stekelenburg and Vroomen, 2005; Teder-Sälejärvi et al., 2005; Yang et al., 2013; Gao et al., 2014; Ren et al., 2018; Zhao et al., 2018, 2020), and the factors responsible for this polarity reversal remain unexplored to date. However, given that these ERP activities, regardless of polarity, have been viewed generally as an effect of auditory signals on early visual discrimination (Giard and Peronnet, 1999; Molholm et al., 2002, 2004; Teder-Sälejärvi et al., 2002; Stekelenburg and Vroomen, 2005; Teder-Sälejärvi et al., 2005; Kaya and Kafaligonul, 2019) and located consistently in the ventral extrastriate visual cortex (Teder-Sälejärvi et al., 2002; Molholm et al., 2004; Teder-Sälejärvi et al., 2005), it is reasonable to hypothesize that the aforementioned N195 and P195 components are different manifestations of the same cross-modal neural activity. Additional research may be required to test this hypothesis further.

One might question that the timing of the high-level congruence-induced facilitation here (i.e., ~200 ms post-stimulus onset) is too early to be true. However, it is noteworthy that many previous studies have reported prominent ERP differences between semantically congruent versus incongruent audiovisual conditions beginning earlier than 200 ms post-stimulus (e.g., Molholm et al., 2004; Stekelenburg and Vroomen, 2007; Liu et al., 2011; Hu et al., 2012), although the directionality of these early congruency effects (congruence-induced vs. incongruence-induced) is indistinguishable. More relevantly, by removing ERPs associated with low-level audiovisual spatiotemporal integration in the processing of semantically congruent audiovisual stimuli, a recent study found that the earliest isolated ERP component purely representing congruence-induced semantic integration was a parieto-occipitally distributed negativity during 220–240 ms (Xi et al., 2020), which is highly analogous to the *difference* in N195 between the current congruent-sound and neutral-sound conditions (i.e., the neural basis of the congruence-induced benefit here). Notably, the observation that these congruence-induced audiovisual neural interactions were mainly located over the occipital scalp is consistent with the well-established role of the occipital cortex in encoding semantically congruent audiovisual objects (Murray et al., 2004; Naghavi et al., 2011). In addition, a more recent study of congruent character-speech integration also found an early ERP difference between their congruent-character and neutral-character conditions during 150–200 ms (Du et al., 2022), although this effect was more anteriorly distributed than the current N195 effect, which could be attributed to the well-known dominance of auditory processing in speech perception (Van Atteveldt et al., 2004). Taken together, prior knowledge in the literature strongly indicates that the early congruence-induced N195 amplitude enhancement here represents a genuine semantic-based audiovisual interaction that would further facilitate the discriminative processing of visual stimuli in addition to the benefit from co-occurring spatiotemporal-based audiovisual interactions.

The N195 component was followed by the late cross-modal difference component N440 over the parietal region (measured during 400–500 ms after the onset of T2 over the electrodes P1, Pz, and P2). This component has been shown to be the neural basis for the limited effect of semantically incongruent sounds on reducing attentional blink (Zhao et al., 2021, 2022). The results of the present study show that the N440 component was significantly evoked only when semantically incongruent sounds accompanied T2 at the same time, but not when semantically neutral or congruent sounds accompanied T2. These findings are fully in line with the findings of Zhao et al. (2021, 2022) that the component was only sensitive to audiovisual stimuli with semantic conflict. Many other previous studies have also found similar N400-like components caused by semantically incongruent audiovisual integration (Molholm et al., 2004; Zimmer et al., 2010; Liu et al., 2011; Donohue et al., 2013; Kang et al., 2018), and these N400 deflections are typically accompanied by slower reaction times and/or lower accuracy in response to semantically incongruent audiovisual pairs (Zimmer et al., 2010; Donohue et al., 2013; Kang et al., 2018). The current incongruence-specific N440 activity and the decreased sound-induced T2 discrimination enhancement in the incongruent-sound condition relative to the neutral-sound condition (i.e., the incongruence-induced behavioral cost) fit well with the association between the N400 component and behavioral performance. Therefore, the present N440 results indicate that during the modulation of audiovisual semantic congruency on the sound-induced alleviation of visual attentional blink, the incongruence-induced cost unfolds at a late stage of processing, where the compatibility of multiple stimuli representations may be evaluated in detail (Lau et al., 2016; Mantegna et al., 2019).

In compliance with previous studies on the meaningless-sound-induced alleviation of visual attentional blink (Olivers and Van der Burg, 2008; Kranczioch and Thorne, 2013, 2015; Kranczioch et al., 2018; Wang et al., 2022), the current study also used a pure tone to represent the meaningless, semantically neutral sound. Notwithstanding, one could argue that because some real-world objects may also make sounds like a pure tone (e.g., a telephone’s call waiting sound), the tone used here might have still been perceived as somewhat semantically incongruent with the T2 stimulus set (i.e., dogs, cars, and drums). However, were this argument tenable, it is highly noteworthy that the incongruence-sensitive N440 activity discussed above should have also been prominent, rather than completely absent, in the neutral-sound condition (see Figure 4D). Thus, it appears that, as expected, the pure tone here was more likely to be perceived as semantically neutral with respect to T2. Having said that, additional research should consider using other types of auditory stimuli (e.g., white noise, scrambled object sound) as the semantically neutral sound and examine whether the current main findings can be generalized.

As the present study successfully separated the differential effects of semantically congruent and incongruent sounds on visual attentional blink as well as their distinct electrophysiological bases, we propose an update to the hierarchical model recently suggested by Zhao et al. (2021) regarding how the low-level and high-level properties of a sound affect T2 processing during the attentional blink. At the *first* stage, a T2-accompanying task-irrelevant sound, regardless of its semantic relevance (congruent, neutral, incongruent) with respect to T2, would strengthen the perceptual processing of T2 through a relatively early, spatiotemporal-based audiovisual neural interaction, which provides an initial foundation for T2 to escape the attentional blink. Importantly, when the sound is semantically congruent with T2, it would further facilitate the perceptual processing of T2 at the same stage through not only the low-level audiovisual interaction but also a co-occurring, semantic-based audiovisual interaction, hence laying a more solid foundation for T2 to escape the attentional blink. At the *second* stage, T2s paired with congruent and neutral sounds would smoothly pass the detailed evaluation of the compatibility of auditory and visual representations and then escape the attentional blink based on the magnitude of perceptual benefit obtained at the previous stage. In contrast, T2 paired with a semantically incongruent sound would be stuck at this stage due to the conflict between auditory and visual representations generating a processing cost, which in turn counterbalances the perceptual benefit acquired at the first stage and thus restrains T2 from escaping the attentional blink. As shown above, the updated model highlights the unique role of semantically congruent audiovisual integration in enhancing the perceptual processing of a visual object, through which the object’s fine characteristics would be more likely to survive the temporal limitation of attention.

As a direction for future research, given that one of the most accepted viewpoints about the attentional blink *per se* considers it as a structural limitation in working memory encoding (Vogel et al., 1998; Petersen and Vangkilde, 2022; for reviews, see Martens and Wyble, 2010; Zivony and Lamy, 2022), additional studies are required to further showcase whether the aforementioned spatiotemporal-based and semantic-based early audiovisual interactions alleviate the visual attentional blink *ultimately by* enhancing working memory encoding. Indeed, this linkage is very likely to be true in light of many previous findings showing that working memory encoding could be substantially enhanced when semantically congruent, or even semantically irrelevant, auditory and visual stimuli are presented synchronously (Delogu et al., 2009; Bigelow and Poremba, 2016; Xie et al., 2017; Aizenman et al., 2018; Almadori et al., 2021; Yu et al., 2022; for an early review, see Mastroberardino et al., 2008). It should be noted that the sound-induced alleviation of visual attentional blink is less likely to result from the sound’s co-occurrence and semantic congruence directly enhancing T2 encoding in working memory without early audiovisual interactions. Instead, the prominent cross-modal N195 activity here, as well as the prior evidence showing that the N195 amplitude was larger preceding correct than incorrect discriminations of audiovisual T2s during the blink (Zhao et al., 2021), strongly suggests that the potential enhancement of working memory encoding, if any, would be mainly the consequence of early audiovisual interactions.

Last but not least, the current findings also have general implications for future studies on audiovisual semantic congruency effects. In the extant literature, the most common approach to tracking semantic-based audiovisual processes has been to target the differences in behavioral and neutral responses between the congruent versus incongruent audiovisual conditions (e.g., Molholm et al., 2004; Stekelenburg and Vroomen, 2007; Zimmer et al., 2010; Liu et al., 2011; Hu et al., 2012; Donohue et al., 2013; Kang et al., 2018). However, by introducing a semantically unrelated audiovisual condition as the baseline, the current results clearly exemplify that this approach has the risk of mixing up congruence-induced benefits and incongruence-induced costs, which could be detrimental to theoretical understandings of how semantic-based multisensory processes operate and affect other cognitive processes. Therefore, the current findings call for the use of semantically unrelated audiovisual combinations as the baseline whenever possible in future studies on audiovisual semantic congruency effects.

## 5 Conclusion

Although recent research has isolated a congruent-sound-induced enhancement of the allocation of *space-based* visual attention by introducing a neutral-sound-accompanying audiovisual condition as the baseline (Maezawa et al., 2022), the current study is the first to demonstrate an unequivocal congruent-sound-induced benefit in alleviating the limitation of *time-based* visual attention indexed by the attentional blink. This congruence-induced behavioral improvement was further accompanied by an enhanced audiovisual neural interaction over the occipital scalp at approximately 200 ms post-stimulus onset, suggesting that it begins at a relatively early stage of visual discrimination. These findings extend our understanding of how audiovisual semantic congruency modulates the sound-induced alleviation of visual attentional blink, and call for an update on the hierarchical model previously proposed by Zhao et al. (2021), in which the congruent-sound-induced contribution was underestimated. More generally, these findings also add to the growing number of studies showing the particular role of semantically congruent multisensory integration in facilitating perception, attention, memory, and so on (Hsiao et al., 2012; Heikkilä and Tiippana, 2016; Heikkilä et al., 2017; Marian et al., 2021; Duarte et al., 2022; Maezawa et al., 2022).

## Data availability statement

The datasets presented in this study can be found in online repositories. The names of the repository/repositories and accession number(s) can be found at: Open Science Framework, https://osf.io/bd845/.

## Ethics statement

The studies involving humans were approved by Institutional Review Board of Soochow University. The studies were conducted in accordance with the local legislation and institutional requirements. The participants provided their written informed consent to participate in this study.

## Author contributions

SZ: Conceptualization, Data curation, Formal analysis, Funding acquisition, Software, Supervision, Visualization, Writing – original draft, Writing – review & editing. YZ: Conceptualization, Data curation, Formal analysis, Investigation, Validation, Visualization, Writing – original draft, Writing – review & editing. FM: Formal analysis, Investigation, Validation, Writing – original draft. JX: Formal analysis, Investigation, Validation, Writing – original draft. CF: Project administration, Resources, Supervision, Validation, Writing – review & editing. WF: Conceptualization, Data curation, Funding acquisition, Project administration, Resources, Supervision, Writing – original draft, Writing – review & editing.

## Footnotes

^1^ https://jasp-stats.org/
