# Supplementary material for: The dissociation of semantically congruent and incongruent cross-modal effects on the visual attentional blink
Source: Front Neurosci. 2023 Dec 14;17:1295010. doi: 10.3389/fnins.2023.1295010 (PMC10755906; doi:10.3389/fnins.2023.1295010)
Supplement: Supplementary file 1 [file Data_Sheet_1.docx]

Supplementary Materials

The dissociation of semantically congruent and incongruent cross-modal effects on the visual attentional blink

Song Zhao^1†^, Yuxin Zhou^1†^, Fangfang Ma^1^, Jimei Xie^1^, Chengzhi Feng^1^, Wenfeng Feng^1,2^*

^1^ Department of Psychology, School of Education, Soochow University, Suzhou, China

^2^ Research Center for Psychology and Behavioral Sciences, Soochow University, Suzhou, China

*** Correspondence:**Wenfeng Feng ([fengwfly@gmail.com](mailto:fengwfly@gmail.com))

^†^ These authors contributed equally to this work and share first authorship

# Rating the representativeness of the line drawings and object sounds

As noted in the main text, the line drawings of common objects (three clothes, three cups and three flowers used as T1; three dogs, three cars and three drums used as T2) and the object sounds (three dog barks, three car beeps and three drumbeats) used in the current study were the same as those used in the Zhao et al. (2021) study. These stimuli are openly available at <https://osf.io/bd845/>. Moreover, all of our participants verbally reported that they could identify, without any difficulty, the object categories of all line drawings and object sounds when these stimuli were presented in isolation before the beginning of the formal experiment, suggesting that these stimuli are well representative of their corresponding object categories as expected.

Nevertheless, to provide quantitative evidence for this proposal and facilitate the use of these materials in the future, here, we recruited an independent group of 30 participants (20 females and 10 males; age range 18–28 years, mean age 22.7 years) to rate the representativeness of these materials. Specifically, for each of these stimuli, participants were first required to ***choose what object category the presented stimulus was representative of from three options*** (for T1 drawings, the options were clothes, cup and flower; for T2 drawings and object sounds, the options were dog, car and drum). Second, participants were required to ***rate the degree to which the presented stimulus was representative of the chosen object category using a 7-point rating scale*** (1 = not at all, 4 = intermediately, 7 = absolutely). The T1 drawings, T2 drawings and object sounds were presented in separate blocks, and the order of the three blocks was counterbalanced across participants. Within each block, the corresponding nine stimuli were also presented in a randomized order.

The results first showed that, as expected, the object categories of all drawings and object sounds were correctly identified by each participant. Second, as shown in Table S1 below, the overall rating score was very high, confirming that these stimuli are well representative of their corresponding object categories. More importantly, the one-way repeated-measures ANOVA revealed that there was no significant difference in rating score among the three different identities (e.g., dog #1, dog #2 and dog #3) under the same object category, and this was true regardless of object category and stimulus set (i.e., T1 drawings, T2 drawings and object sounds). Thus, these results demonstrate that under each object category, the three different identities are equally representative of their corresponding object category.

**Table S1**. Group-mean rating scores (*n* = 30) of the degree to which the stimulus materials were representative of their corresponding object categories, and comparison of rating scores among three different identities under each object category.

| **Stimulus set** | **Object category** | **Identity** | **Rating score** | | ***F***(2, 58) | ***p*** | ***η²***p | BF10 |
| --- | --- | --- | --- | --- | --- | --- | --- | --- |
|  |  |  | ***M*** | ***SD*** |  |  |  |  |
| T1 drawings | Clothes | #1 | 6.967 | 0.183 | 1.526 | 0.226 | 0.050 | 0.340 |
|  |  | #2 | 6.933 | 0.254 |  |  |  |  |
|  |  | #3 | 7.000 | 0.000 |  |  |  |  |
|  | Cup | #1 | 6.967 | 0.183 | 1.420 | 0.250 | 0.047 | 0.300 |
|  |  | #2 | 6.900 | 0.403 |  |  |  |  |
|  |  | #3 | 6.867 | 0.346 |  |  |  |  |
|  | Flower | #1 | 6.833 | 0.531 | 0.048 | 0.953 | 0.002 | 0.103 |
|  |  | #2 | 6.867 | 0.507 |  |  |  |  |
|  |  | #3 | 6.833 | 0.648 |  |  |  |  |
| T2 drawings | Dog | #1 | 6.967 | 0.183 | 1.000 | 0.374 | 0.033 | 0.214 |
|  |  | #2 | 7.000 | 0.000 |  |  |  |  |
|  |  | #3 | 6.967 | 0.183 |  |  |  |  |
|  | Car | #1 | 7.000 | 0.000 | 1.000 | 0.374 | 0.033 | 0.250 |
|  |  | #2 | 6.967 | 0.183 |  |  |  |  |
|  |  | #3 | 7.000 | 0.000 |  |  |  |  |
|  | Drum | #1 | 7.000 | 0.000 | 2.071 | 0.135 | 0.067 | 0.661 |
|  |  | #2 | 6.933 | 0.254 |  |  |  |  |
|  |  | #3 | 7.000 | 0.000 |  |  |  |  |
| Object sounds | Dog bark | #1 | 6.933 | 0.254 | 1.650 | 0.201 | 0.054 | 0.393 |
|  |  | #2 | 6.800 | 0.664 |  |  |  |  |
|  |  | #3 | 6.967 | 0.183 |  |  |  |  |
|  | Car beep | #1 | 6.800 | 0.484 | 1.374 | 0.261 | 0.045 | 0.364 |
|  |  | #2 | 6.900 | 0.403 |  |  |  |  |
|  |  | #3 | 6.967 | 0.183 |  |  |  |  |
|  | Drumbeat | #1 | 6.767 | 0.504 | 2.243 | 0.115 | 0.072 | 0.572 |
|  |  | #2 | 6.700 | 0.651 |  |  |  |  |
|  |  | #3 | 6.900 | 0.305 |  |  |  |  |

# References

Zhao, S., Feng, C., Huang, X., Wang, Y., and Feng, W. (2021). Neural basis of semantically dependent and independent cross-modal boosts on the attentional blink. *Cereb. Cortex 31*, 2291–2304. doi: 10.1093/cercor/bhaa362
